# Spatial and spectral structure of local functional connectivity of the background intracranial EEG in patients with focal epilepsy

**DOI:** 10.3389/fnetp.2025.1441949

**Published:** 2026-01-09

**Authors:** Hitten P. Zaveri, Steven M. Pincus, Irina I. Goncharova, Reshma Munbodh, Lawrence J. Hirsch, Robert B. Duckrow, Dennis D. Spencer

**Affiliations:** 1 Department of Neurology, Yale University, New Haven, CT, United States; 2 Yale University, Guilford, CT, United States; 3 Department of Pediatrics, Yale University, New Haven, CT, United States; 4 Department of Neurosurgery, Yale University, New Haven, CT, United States

**Keywords:** brain networks, coherence, connectivity, epilepsy surgery outcome, seizure onset area

## Abstract

**Purpose:**

To determine the frequency band-related local functional connectivity (BRLFC) of the seizure onset area (SOA) and areas removed from it, and the relationship between BRLFC and outcome of epilepsy surgery.

**Methods:**

This study was conducted on 14 unselected adult patients with focal epilepsy undergoing icEEG monitoring for surgery. Intracranial EEG (icEEG) electrode contacts were located from post-implantation CT and MR images and registered to the MRI of a common brain to allow interpretation of results from all patients in the same space. Two 1 h icEEG epochs, recorded during wake and removed in time from seizure occurrence, were studied. One of these epochs was when the subject was on anti-seizure medications (ASMs), while the second was after ASM taper. Coherence was estimated for all pairs of electrode contacts ipsilateral to the SOA in delta, theta, alpha, beta, gamma and a high frequency band. The BRLFC of each electrode contact was estimated as the average band-related coherence between it and all electrode contacts within a spatial window.

**Key findings:**

BRLFC in the SOA and peri-SOA, for selected frequency bands, was greater in patients with excellent outcome after surgery in comparison to those with poor outcome. A graded relationship was observed between BRLFC and distance to the SOA of patients with excellent outcome to surgery such that contacts with the greatest connectivity were closer to the SOA and those with the lowest connectivity were several cm from the SOA. This relationship between distance to the SOA and connectivity was present primarily in the alpha, beta, gamma and high frequency bands and the BRLFC was greatest in the peri-SOA, within a distance of 5 cm from the SOA. This relationship was stable between on-ASMs and off-ASMs epochs.

**Significance:**

There is stable altered BRLFC in the SOA and peri-SOA expressed in the background icEEG of patients with focal epilepsy. This altered BRLFC may be a network marker of medically intractable focal epilepsy which is related to outcome of epilepsy surgery.

## 1 Introduction

The pre-surgical evaluation of patients with focal epilepsy, in whom seizures cannot be controlled by medication, involves a series of increasingly more invasive investigations, and may require, as a final step, continuous monitoring of intracranial EEG (icEEG) to locate the seizure onset area (SOA). During icEEG monitoring seizures are recorded over days to weeks. The SOA is located by visual analysis of the icEEG. There has also been interest in the localization of the SOA through computer-assisted analysis of ictal and interictal brain activity. Several recent studies have used network-based analysis of the magnetoencephalogram (MEG) or EEG. These studies have measured the correlation of interictal activity at electrode contacts seeking to locate the SOA, relate this to outcome of epilepsy surgery and further our understanding of focal epilepsy as a network disorder (Spencer, 2002).

Studies have reported a considerable change in functional networks in focal epilepsies. Evaluations of EEG connectivity has been performed to a greater extent for studies of seizures, seizure spread and seizure prediction (Spencer, 2002; Bartolomei et al., 2017; Bialonski and Lehnertz, 2013; Brazier, 1972; Brazier et al., 1972; Brazier and Brazier, 1973; Burns et al., 2014; Dickten et al., 2016; Geier and Lehnertz, 2017; Gotman, 1983; Gotman, 1987; Guye et al., 2006; Khambhati et al., 2016; Kramer et al., 2008; Kramer et al., 2012; Lieb et al., 1987a; Lieb et al., 1987b; Netoff and Schiff, 2002; Rings and Lehnertz, 2016; Schindler et al., 2008; Smith et al., 2016; Sritharan and Sarma, 2014; Terry et al., 2012; Varotto et al., 2012; Wendling et al., 2010; Wilke et al., 2011), and to a lesser extent on the interictal state (Bartolomei et al., 2017; Dickten et al., 2016; Geier and Lehnertz, 2017; Varotto et al., 2012; Wilke et al., 2011; Palmigiano et al., 2012; Zaveri et al., 2009a; Warren et al., 2010; Tomlinson et al., 2017; Sinha et al., 2017; Bettus et al., 2008; Lee et al., 2014; Constable et al., 2013; Nissen et al., 2017; Englot et al., 2015; Park and Madsen, 2018; Wang et al., 2020; Stone et al., 2022). Studies show increased relationships at the start of a seizure, and considerable change during the seizure, likely reflecting networks involved in seizure propagation, the recruitment of additional brain regions, seizure maintenance and seizure termination (Bialonski and Lehnertz, 2013; Khambhati et al., 2016; Chu et al., 2015; Yaffe et al., 2015; Smith and Schevon, 2016; Lehnertz et al., 2023). The networks revealed by EEG based approaches appear to be large scale networks, like the resting state networks (RSNs) shown through fMRI analysis. Studies have sought to analyze and model the changes during a seizure (Smith et al., 2016; Jiruska et al., 2013) as synchronizing and de-synchronizing dynamics (Khambhati et al., 2016) or by resolving distinct brain network states (Burns et al., 2014; Sritharan and Sarma, 2014; Yaffe et al., 2015). These approaches provide insight into the SOA and its influence on the evolving seizure. While these studies are important, many networks may be activated during a seizure and EEG synchrony during this state may reflect multiple mechanisms (Frei et al., 2010). Further, many of the methods used for determination of connectivity, such as correlation, assume signal stationarity, which is unlikely during a seizure (Frei et al., 2010; Zaveri et al., 1999). Thus, network analysis during seizure to locate the SOA may be more challenging, indicating that other, more robust approaches are required.

The interictal state has revealed aberrant connectivity related to the SOA and surgical outcome. Studies of interictal connectivity have been conducted with intracranial EEG (icEEG) (Bartolomei et al., 2017; Dickten et al., 2016; Geier and Lehnertz, 2017; Varotto et al., 2012; Wilke et al., 2011; Palmigiano et al., 2012; Zaveri et al., 2009a; Warren et al., 2010; Tomlinson et al., 2017; Sinha et al., 2017; Bettus et al., 2008; Park and Madsen, 2018; Wang et al., 2020; Stone et al., 2022), fMRI, (Lee et al., 2014; Constable et al., 2013), and MEG (Nissen et al., 2017; Englot et al., 2015) based on the argument that an epileptic network should be persistently abnormal between seizures and could therefore also be defined by the extent and strength of its interictal components and connections (Spencer et al., 2018). Tomlinson and colleagues, for example, used a machine learning method combining global connectivity, root-mean-square of the EEG amplitude, and power in the delta band of 1,200 randomly selected 1-s icEEG segments in each of 17 pediatric patients, 15 with focal cortical dysplasia. They observed increased global synchrony in patients who fared poorly after surgery and predicted surgical outcome in 16 of 17 patients (Tomlinson et al., 2017). Sinha and colleagues used a correlation-based measure of 1 h interictal icEEG epochs within a computational model of a bistable (interictal and seizure states) network to propose a resection area and determine the overlap between the proposed resection area and the clinically identified SOA. The method correctly classified patients with good outcome in 7/8 cases and correctly classified surgical failure in 6/8 cases (Sinha et al., 2017). As indicated in a commentary (Eissa and Schevon, 2017), this study (Sinha et al., 2017) is suggestive of the value of functional connectivity analysis of the interictal EEG, but also underscores the work which remains to be performed to better understand the information which is being reported on functional connectivity of the interictal EEG.

While there has been an increasing interest in the network basis of epilepsy and seizures, the nature of the network, its spatial extent and how the network’s behavior is expressed in electrophysiology remain unknown. The relation of the SOA to the seizure generating network also remains unknown. In previous studies, we demonstrated that the SOA and areas which are spatially removed from it have non-zero band-related local functional connectivity (BRLFC) in the beta frequency band (Zaveri et al., 2009a; Zaveri et al., 2008). In these studies, we used BRLFC, a measure of the connectivity of an electrode contact to all electrode contacts within a spatial window, rather than focus on just the relationship between pairs of electrode contacts. In this study, we compared the BRLFC of patients with excellent outcome (EO) and poor outcome (PO) to epilepsy surgery, for different frequency bands at two time points during icEEG monitoring, when patients were on anti-seizure medications (on-ASMs) and off-ASMs. We demonstrate that there are differences in BRLFC in selected frequency bands in EO and PO patients. Further, in EO patients BRLFC, in some frequency bands, demonstrates a graded function of distance from the SOA such that relatively higher values are observed in the peri-SOA and lower values are observed at a distance from the SOA. In PO patients, in contrast, the same spatial profile is not observed. We demonstrate that the BRLFC differences and spatial profiles are temporally stable over days with medication taper. We propose a two-part description of the BRLFC, a decomposition into low-frequency and high frequency bands, based on the spatial and spectral structure of the BRLFC in the EO and PO groups. We demonstrate that it is possible to distinguish EO and PO patients based on the values of low-frequency and high-frequency BRLFC. These observations have implication for efforts to understand medically intractable focal epilepsy, the network theory of epilepsy, the SOA and peri-SOA areas and predict the outcome of epilepsy surgery.

## 2 Methods

### 2.1 Subjects

Subjects were 14 consecutive adult patients undergoing icEEG monitoring for epilepsy surgery, where the intracranial monitoring was free of complications, the SOA could be identified and was unilateral and the patients proceeded to surgery. The average age of the subjects was 33.29 years and 6 of the subjects were female. Five of the subjects had medial temporal onset and 9 had neocortical seizure onset. The subjects were placed into two groups based on outcome to surgery evaluated at a consistent endpoint of 5 years for all subjects: excellent outcome (EO, Engel’s class 1 or 2), and poor outcome (PO, Engel’s class 3 or 4). Eight patients had excellent outcome and 6 had poor outcome. Three of the 5 medial temporal onset patients and 5 of the 9 neocortical onset patients had excellent outcome. The age, gender, location of the SOA, areas monitored with icEEG and outcome to surgery for the subjects are listed in Table 1.

**TABLE 1:** The gender, age, number of icEEG contacts, brain areas sampled by icEEG contacts, location of the SOA and outcome to surgery for the subjects are listed.

Patient	Gender	Age	Contacts	Brain areas sampled	Seizure onset location	Outcome
1	M	20	218	IT, LT, O, P, F	L anterior medial frontal	EO
2	M	51	174	IT, LT, O, P, F, MT	L medial temporal	EO
3	F	35	113	IT, LT, O, P, F	L superior parietal	PO
4	M	26	203	IT, LT, O, P, F, MT	L medial temporal	EO
5	M	27	100	IT, LT, O, P, F, MT	R anterior superior lateral temporal	PO
6	M	23	174	IT, LT, O, P, F, MT	R temporo-parietal	PO
7	M	54	220	IT, LT, O, P, F, MT	R inferior temporal	PO
8	F	27	261	IT, LT, O, P, F, MT	R medial temporal	PO
9	F	41	201	IT, LT, O, P, F, MT	R parietal	EO
10	M	31	146	IT, LT, o, P, F, MT	R inferior temporal	EO
11	F	28	228	IT, LT, O, P, F, MT	R medial temporal	PO
12	F	39	216	IT, LT, O, P, F, MT	L medial temporal	EO
13	F	26	194	IT, LT, o, P, F, MT	R parietal	EO
14	M	38	252	IT, LT, o, P, F, MT	L post inferior temporal	EO

Eight subjects had excellent outcome (EO), five with left hemisphere onset, and six had poor outcome (PO), one with left hemisphere onset. Five of the subjects had medial temporal onset of seizures, three of whom had excellent outcome. Nine of the subjects had neocortical seizure onset five of whom had excellent outcome. The ipsilateral brain areas sampled were inferior temporal (IT), lateral temporal (LT), medial temporal (MT), occipital (O), parietal (P), and frontal (F).

### 2.2 Intracranial electrode placement and localization

Between 100 and 261 electrode contacts were placed in the 14 subjects (median 202). Most of the electrode contacts were subdural strip or grid electrode contacts and relatively few were depth electrode contacts. To determine the location of electrode contacts, individual contacts were marked on postoperative CT scans. CT scans were first co-registered to post-operative MRI scans using a 6-parameter rigid transformation. The post-operative MRI scans were then co-registered with pre-operative scans using a nonlinear grid-based transformation which used normalized mutual information as the similarity metric to account for the distortion of the brain that may occur due to craniotomy (Papademetris et al., 2004; Skrinjar, 2002). This procedure has been followed at our institute for several hundred patients as part of icEEG monitoring for epilepsy surgery. The pre-operative MRI was subsequently co-registered with the MRI of a common brain (Holmes et al., 1998) using a second, similar, non-linear transform and electrode contacts were located on the common brain. Intercontact distance was measured as Euclidean distance using the 3D coordinates of the electrode contacts on the common brain.

The SOA was identified by a standing multidisciplinary team in the Yale Comprehensive Epilepsy Center using standard clinical criteria. The number of seizure onset contacts, that is the number of contacts in the SOA, ranged from 1 to 6 in the 14 subjects. The study was limited to intracranial electrode contacts ipsilateral to the SOA. The distance of each electrode contact from the SOA was determined as the distance from the contact to the closest SOA contact. The distance of a seizure onset contact to the SOA was defined to be zero.

### 2.3 Intracranial EEG acquisition

Up to 128 channels of icEEG were each sampled at 256 Hz and recorded along with time synchronized video and audio of the patient (Natus Medical Inc./Bio-logic Systems Corp., San Carlos, California). Intracranial EEGs were recorded with respect to a peg electrode placed within the skull at a distance from the icEEG electrode contacts, and a contact on an inverted strip electrode placed in subgaleal space was used as the ground electrode. The entire icEEG monitoring period was recorded for the 14 subjects. Subsequently we identified two 1-h epochs, one during an on-ASMs time period, from day 2 or 3 of the monitoring, and the second after ASMs had been tapered (Goncharova et al., 2016; Goncharova et al., 2013; Zaveri et al., 2010; Zaveri et al., 2009b; Goncharova et al., 2009; Spencer et al., 2008). The epochs were at least 6 h from a seizure and when the patient was awake and resting quietly. The time period was identified after an examination of medication records, seizure times, and the video and visual inspection of the icEEG. An attempt was made to identify the 1 h on-ASMs epoch during the morning of day 2 or 3. If a suitable epoch could not be identified during the morning we selected an epoch during the afternoon. The off-ASMs epoch was matched to the on-ASMs epoch in terms of time of day. The ASMs typically used, their taper and the impact of the taper on the icEEG can be found in our previous studies (Goncharova et al., 2016; Goncharova et al., 2013; Zaveri et al., 2010; Zaveri et al., 2009b; Goncharova et al., 2009; Spencer et al., 2008).

### 2.4 Estimating coherence and band related local functional connectivity

Coherence was estimated by the weighted-overlapped segment averaging (WOSA) coherence estimator (Carter, 1987; Zaveri et al., 1999) for all pairs of electrode contacts for the delta (0–4 Hz), theta (4–8 Hz), alpha (8–13 Hz), beta (13–25 Hz), gamma (25–55 Hz) and high (65–128 Hz) frequency bands. To estimate the coherence, icEEG epochs were segmented (segment length T = 1 s, successive signal segments were not overlapped), the mean of each signal segment was deleted, and the segments were weighted with a Hann window before calculation of the fast Fourier transform (Zaveri et al., 1999). The magnitude squared coherence was calculated from the coherence estimates (Zaveri et al., 1999; Carter et al., 1973). A referential montage was used. Checks were performed to ensure a lack of reference contamination (Zaveri et al., 2000).

An examination of MSC estimates demonstrate a strong dependence on the distance between the pair of electrode contacts, with most estimates decreasing within 5 cm to lower values (Supplementary Figure 1). We were interested in a measure of functional connectivity which was local to the electrode contact and chose to focus on a maximum intercontact distance of 5 cm to estimate BRLFC. This was the spatial window selected for our estimate of the BRLFC for this study. This decision was based on our interest in local functional connectivity (LFC) and the observed profile of MSC estimates as a function of the inter-contact distance. We define the BRLFC of an electrode contact *p*, as *C(p)*, and estimate it as the average magnitude squared coherence (MSC), for a given frequency band of interest, of electrode contact *p* and all other ipsilateral electrode contacts within the BRLFC spatial window.

The electrode contacts ipsilateral to the SOA were separated into three sets based on distance from the SOA: 1) *S*: electrode contacts within the SOA, 2) *P*: all electrode contacts in the peri-SOA; within 5 cm of the SOA but not including the SOA contacts, and 3) *D*: all distant electrode contacts; electrode contacts between 6 and 10 cm of the SOA. All the electrode contacts studied were defined as a fourth set of electrode contacts, *A* = *{S*, *P*, *D}*. We denote the BRLFC of these areas in the same manner as specified above, for example, the BRLFC of the SOA is denoted as *C(S)* and the BRLFC for the peri-seizure area is denoted as *C(P)*.

### 2.5 Statistical evaluation

The BRLFC in EO and PO groups was compared using the Student’s t-test. This test was restricted to an evaluation of all contacts (*A*), and the results were corrected for multiple comparisons. The BRLFC of electrode contacts in sets *S, P*, and *D* was compared with the Student’s t-test. These comparisons were performed for selected frequency bands. Statistical significance was defined for p < 0.05 with this value being adjusted using Bonferroni correction for the multiple tests which were performed.

## 3 Results

**FIGURE 1:**
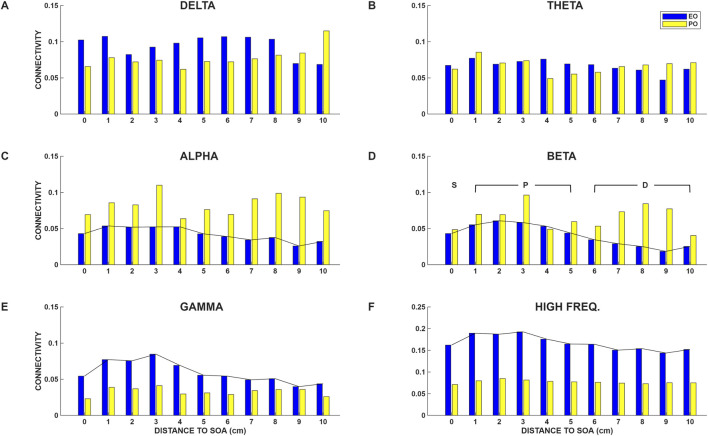
Band-related functional connectivity (BRLFC), averaged over contacts from all subjects, in the 6 frequency bands analyzed are shown for the excellent outcome (EO) and poor outcome (PO) groups, and presented as a function of distance to the seizure onset area (SOA) for the on-ASMs epoch: **(A)** delta, **(B)** theta, **(C)** alpha, **(D)** beta, **(E)** gamma and **(F)** high frequency bands. Each value plotted represents the average connectivity, **(C)** at that distance from the SOA. Considerable difference between EO and PO groups is apparent, particularly in some frequency bands. In the EO group, connectivity displays a graded relationship in the higher frequency bands (alpha through high-frequency bands, see **(C–F)** with distance from the SOA such that greatest connectivity is observed in and around the SOA and lowest connectivity is observed at a distance from the SOA. This graded relationship is not observed in the PO group or in the delta or theta bands in the EO group. S is the seizure onset area, P is the peri-seizure onset area (≤5 cm from the SOA), and D is a distant area (>5 cm from the SOA).

The distance of each electrode contact from the SOA, for all the subjects, was converted to the nearest integer value and the BRLFC values of electrode contacts for each integer distance bin were averaged over all electrode contacts at that distance. This was performed for the on-ASMs data. These average measures are shown in Figure 1 separately for each frequency band and separately for the excellent outcome and poor outcome patients. In Figure 1, the value shown at distance 0 is *C(S)*, the average of the connectivity estimates of all SOA contacts. The estimate plotted at distance 1 is the average connectivity of all electrode contacts at a distance of 1 cm from the SOA, and so on. We note, though these values are low, that there appears to be a difference in the BRLFC of excellent outcome and poor outcome patients in a few frequency bands, and at certain distances from the SOA. Further, there appears to be a graded relationship between average connectivity and distance to the SOA in the alpha, beta, gamma and high-frequency bands in excellent outcome patients. This graded relationship is not apparent in the delta and theta frequency bands. Here, the connectivity estimates at distances of 1–5 cm from the SOA are the largest measured while slightly lower estimates are observed at the SOA. That is, in EO subjects the estimates of connectivity obtained for the SOA are not the highest values observed. The highest values are observed outside the SOA. Further, with increasing distance from the SOA there is a decrease in connectivity values. Examples of the BRLFC of selected individual patients can be found in our previous reports (Zaveri et al., 2009a; Zaveri et al., 2008).

**FIGURE 2:**
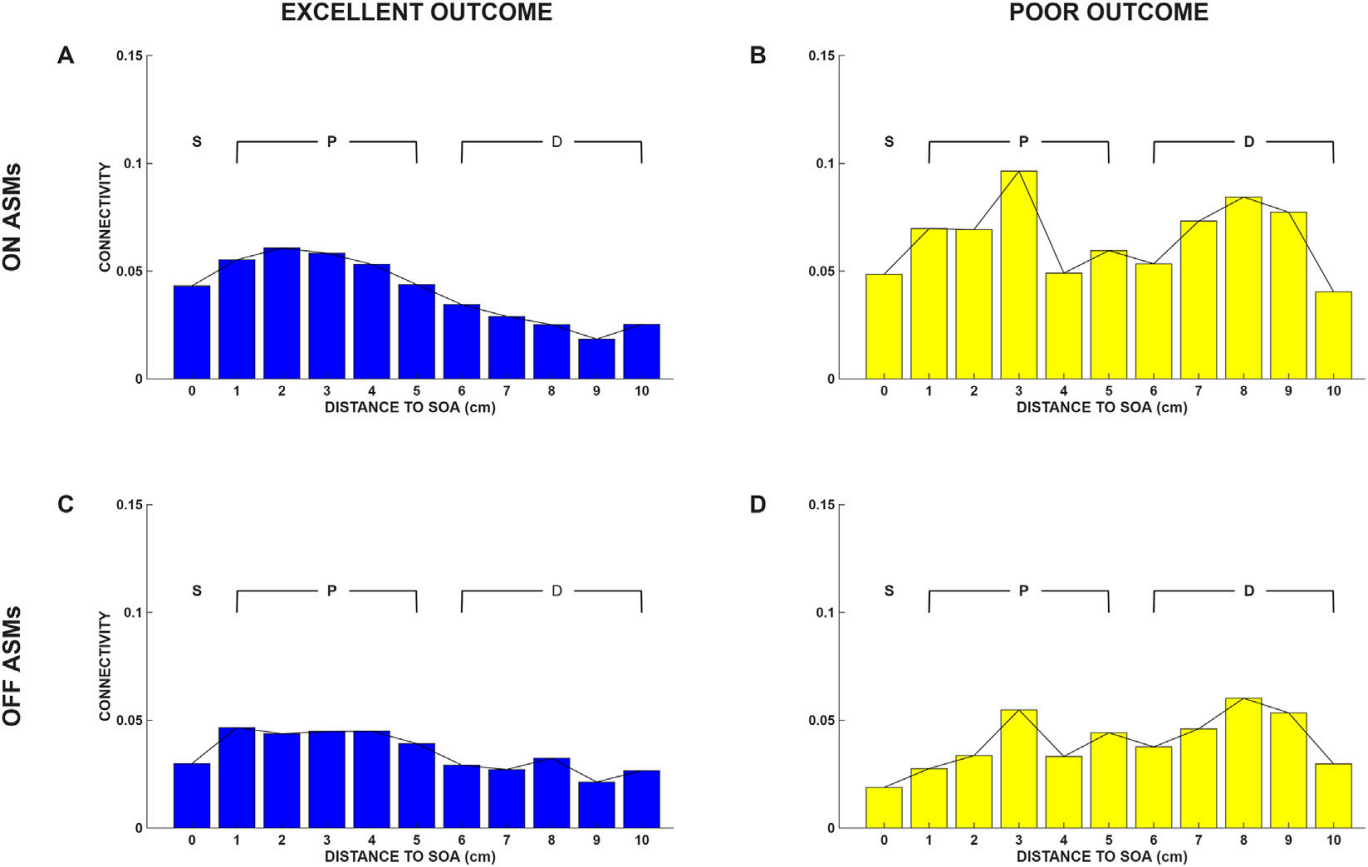
Beta band local functional connectivity in the excellent outcome (EO) and poor outcome (PO) groups, presented as a function of distance to the SOA for both the on- and off-ASMs epochs. The BRLFC estimates for the on-ASMs epoch are shown in the top row for the **(A)** EO and **(B)** PO groups, and for the off-ASMs epoch in the bottom row for the **(C)** EO and **(D)** PO groups. Each value plotted is the average connectivity, C, at that distance from the SOA. This figure is like Figure 1 but evaluated for the beta band and the on- and off-ASMs epochs. In each patient group a similar spatial profile is observed in the off-ASMs period as for the on-ASMs period, suggesting temporal stability over days. S is the seizure onset area, P is the peri-seizure onset area (<= 5cm from the SOA), and D is a distant area (> 5 cm from the SOA).

We next evaluated the BRLFC for the off-ASMs periods. The on-ASMs and off-ASMs BRLFC, for EO and PO subjects are shown for the beta band in Figure 2. The results are presented in a similar manner to those displayed in Figure 1, except that we now display the results only for the beta band and separately for the on-ASMs and off-ASMs epochs. We note that the on-ASMs and off-ASMs evaluations are similar. Though slightly lower values are observed at some distances in the off-ASMs epoch compared to the on-ASMs epoch, the above-mentioned profiles with respect to distance from the SOA remain similar in the off-ASMs epoch, and the difference between on- and off-ASMs epochs are not considerable.

The display of BRLFC at different distances to the SOA, presented in Figures 1, 2 suggests the following: 1) there may be a frequency band and distance specific difference between excellent outcome patients and poor outcome patients, and 2) there may be a graded relationship between BRLFC in high-frequency bands and distance to the SOA in EO subjects but not in PO subjects. These observations are important because they imply both a spatial and spectral structure of connectivity in EO subjects, and that the presence of this structure may be linked to surgical outcome.

**FIGURE 3:**
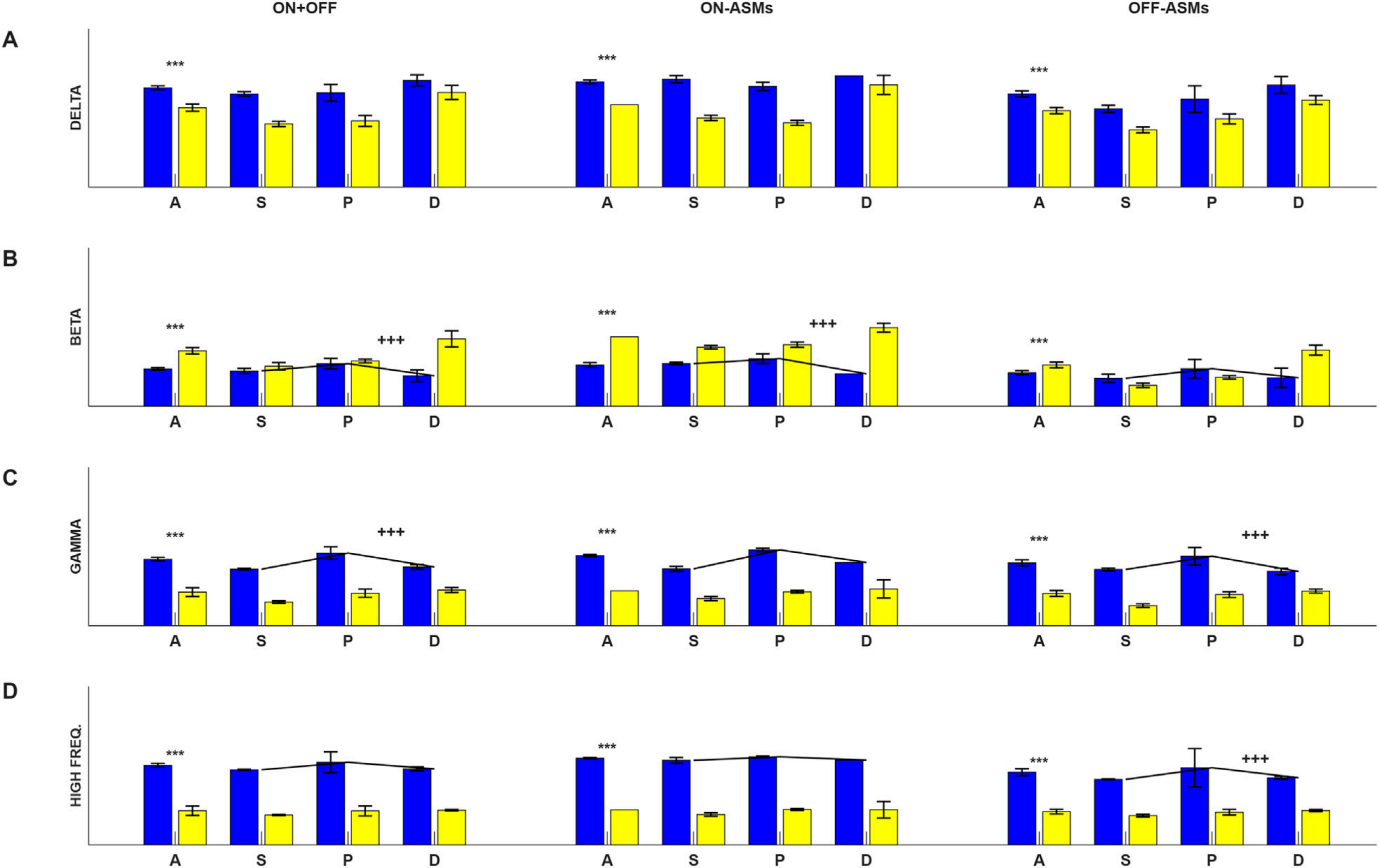
The mean connectivity and the standard error of the mean is displayed for the **(A)** delta, **(B)** beta, **(C)** gamma and **(D)** high frequency bands. The color code is the same as that for Figures 1, 2 (EO = blue, PO = yellow). Data were collected at two times, on-ASMs, and off-ASMs. The three columns display results of: (1) combined on- and off-ASMs data (ON+OFF), (2) on-ASMs, and (3) off-ASMs epochs. The relationships were decomposed by frequency band and distance from the SOA, and then grouped in A (all contacts), S (SOA), P (peri-SOA), and D (distant). A comparison of EO and PO groups for all electrode contacts (A) revealed a difference (significant after Bonferroni correction) in delta, beta, gamma and high-frequency bands for all three data sets (this observation is marked with ‘***’). For these four frequency bands, we tested the observation that connectivity had a graded relationship with distance in the EO group. That is, we tested if C(P) > C(S) and C(P) > C(D). The tests which were significant after correction for multiple comparisons are further marked with ‘+++’.

We next tested these observations. This test was performed for three different datasets: on-ASMs, off-ASMs and a combination of the on- and off-ASM datasets. We first compared the connectivity of EO and PO groups for all the electrode contacts *(A)*. A comparison of EO and PO groups for all electrode contacts revealed a difference (significant after Bonferroni correction) in delta, beta, gamma and high frequency bands for all three data sets (this observation is marked with “***” in Figure 3). For these bands, in the EO group, we next tested the observation that connectivity had a graded relationship with distance. That is, we tested if *C(P)* > *C(S)* and *C(P)* > *C(D)*. This profile was observed in the beta, gamma and high frequency bands, though not consistently in all three datasets. The tests which were significant after correction for multiple comparisons are further marked with ‘+++’ in Figure 3.

**FIGURE 4:**
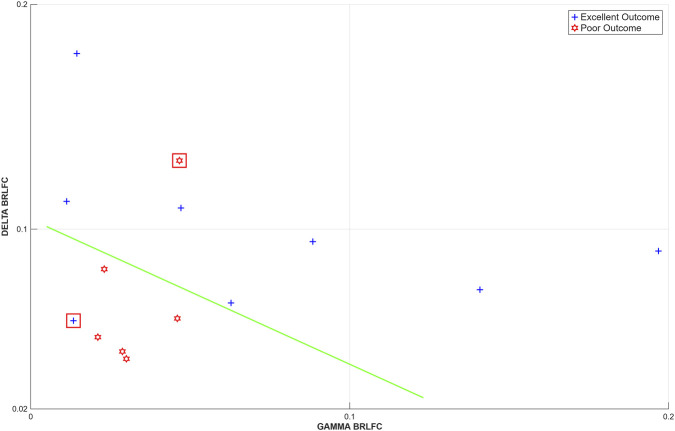
A linear classifier (green line) for separating EO and PO patients based on delta and gamma local functional connectivity in the peri-SOA (P). Greater estimates of delta and gamma connectivity are predictive of excellent outcome while lower estimates are predictive of poorer outcome. One patient each (highlighted) in the EO and PO groups was misclassified.

The results in Figures 1–3 indicate that primary difference in BRLFC between EO and PO patients is in the delta, beta, gamma and high frequency bands. Further, in EO patients *C(P) > C(S)* and *C(P) > C(D)* in selected high frequency bands. These observations are further supported by the use of a classifier built on BRLFC estimates in the delta and gamma bands in the P area which correctly classified 12/14 patients, misclassifying one patient each in the EO and PO groups (see Figure 4). We performed a leave-one-out evaluation of this classifier. In this approach, the data from one subject is left out, and a classifier is built with data from the remaining 13 subjects. The data from the left-out subject, is then tested on the classifier built from the data from the other 13 subjects. Either 1 or 2 patients were misclassified in the 14 leave-one-out tests performed.

## 4 Discussion

Intracranial EEG studies of synchrony and networks conducted with bivariate measures have been used to study seizures and the peri-seizure interval, typically in studies of seizure prediction, seizure onset, and seizure spread, and interictal or background icEEG. In a study of the background icEEG we previously demonstrated increased long-range theta coherence, between amygdala and the frontal lobe, accompanies the process of kindling in a rat model of epilepsy (Blumenfeld et al., 2007). In related studies, Schevon and coworkers demonstrated that local hypersynchrony was a marker of epileptogenic cortex (Schevon et al., 2007). This analysis was performed with the mean phase coherence method (Mormann et al., 2000) for adjacent contacts of a subdural electrode grid. In other studies Staniek and Lehnertz were able to determine the hemisphere of seizure onset with a measure of symbolic transfer entropy (Staniek and Lehnertz, 2008) and Kramer and co-workers employed network measures to demonstrate local and global changes in connectivity at seizure onset (Kramer et al., 2008) Further, Towle and coworkers, used the coherence of the background icEEG to define the functional borders of brain regions (Towle et al., 1998).

Epilepsy is a brain disorder which is expressed by intermittent seizures. In localization-related epilepsy the pathology which expresses the seizures is considered to be focal in nature. The results of this study suggest a re-examination of these two aspects of our understanding of localization-related epilepsy. First, this study demonstrates the presence of non-zero LFC, in multiple frequency bands, at distances of several cm removed from the SOA. Second, this study shows that high functional connectivity exists in the background icEEG at a time removed from a seizure. We note that we selected epochs which were at least 6 h removed from a seizure and at two distinct time-points during intracranial monitoring. This suggests, though seizures, the primary manifestation of this disorder, are expressed intermittently, there is a persistent detectable pathological expression related to the SOA in the background icEEG hours and possibly days before seizure. Second, the results indicate value in a decomposition of the LFC based on frequency and distance from the SOA. Low-frequency LFC (in the delta and theta bands) is distinct from high frequency LFC (alpha through high frequency bands). Third, our results demonstrate a distance and frequency specific increased connectivity in the SOA and peri-SOA which is related to surgical outcome. This suggests there may be critical components or sub-networks of a seizure generating network which are disrupted by surgery in the excellent outcome group. That no such target is observed in the poorer outcome group could suggest that the localization of the SOA in this group was more broadly distributed or erroneous. The classification results indicate a separability between EO and PO patients based on a frequency band and distance specific measure of connectivity.

This study has several limitations. The sample size was small, which restricted the range of statistical analyses that could be conducted. Participants were unselected and reflect the distribution which may be observed at a surgical center such as ours. For instance, five of the six subjects with poor outcomes had right-hemisphere onset. These preliminary observations require confirmation in a larger patient cohort. It may be possible that the preliminary classification result obtained here from an unselected set of patients could be improved with a more homogeneous set of patients (in terms of etiology or region of onset) and the incorporation of other interictal measures and information from pre-surgical evaluation. We did not establish if areas with high BRLFC were fully or substantially resected during the surgical procedure. This limits our fuller understanding of the value of this measure in determinations such as the classifier shown in Figure 4. This can also be addressed in future studies. Future studies should also include a better description of the SOA and the underlying pathology, i.e., a more refined description of the SOA to determine the difference in BRLFC, for example, in cortical dysplasia, tumor, gliosis, and mesial temporal sclerosis.

We have demonstrated the presence of a relationship between BRLFC and distance to the SOA and between BRLFC and surgical outcome. This suggests that the estimates of BRLFC of the interictal icEEG are robust to the approximations and limitations of our approach.

We believe the spectral and spatial profile of LFC in EO patients displayed here may reflect components of networks which are involved in seizure initiation, propagation, maintenance and termination. The connectivity signatures of these network components possibly emerge through the analysis of 1-h epochs of the background icEEG because of their repeated sub-threshold activation during this time and because these networks have been strengthened through repeated seizures leading to a greater representation of these relationships in ongoing activity. It is possible that BRLFC may hold promise for use in different manners. BRLFC, for example, may hold promise for supporting the determination of the SOA. The SOA is currently located by recording seizures and evaluating icEEG changes at seizure onset. It may be possible to design semi-automated or automated algorithms to aid the determination of the SOA using the BRLFC. For examples of how this could be approached see Figure 3; Supplementary Figure S1 of our previous review (Spencer et al., 2018). If BRLFC is a biomarker for the SOA it may hold value as a quantitative measure of network dysfunction which could be used more broadly in epilepsy (Sinha et al., 2022). If for, for example, it could be evaluated after an injury to the brain, we could follow changes in BRLFC to determine risk of developing epilepsy. That is, it may hold value for tracking epileptogenesis. Similarly, if it is found to inform on seizure occurrence it may have value for understanding seizure generation, forecasting and control (Lehnertz et al., 2023; Kuhlmann et al., 2018; Andrzejak et al., 2023; Zaveri et al., 2020). The BRLFC measure used here may also hold value for the determination of areas with abnormal connectivity in other brain disorders. The full extent of these observations remains to be studied and confirmed. Till this is done we will not know which patient subgroups best demonstrate the effect demonstrated here, and the manner in which it is influenced by the various underlying substrates responsible for epileptogenesis.

## 5 Conclusion

Functional connectivity estimated from background icEEGs shows a graded relationship with distance to the SOA in EO patients such that greater connectivity exists in a high frequency band in a peri-SOA area close to the SOA and lower connectivity exists far from it. This relationship was observed in evaluations of 1 h of background icEEG removed in time from seizure onset, with coherence, a linear measure of signal relationship. Furthermore, the delta and gamma band connectivity was greater in patients who were free of seizures after surgery than those who were not. The results reported in this study suggest there is a continual pathological expression in the background icEEG of subjects with localization related epilepsy reflecting altered functional connectivity at distances both near and far from the SOA. These connectivity markers could reflect aspects of the networks which are involved in seizure generation and propagation. The demonstration of a graded change of this abnormal expression with respect to distance from the SOA may hold promise for better definition of the SOA and a better delineation of cortical areas which contribute to its dysfunction.

## Data Availability

The data analyzed in this study is subject to the following licenses/restrictions: these data cannot be shared without approval of the Yale University Human Investigation Committee and funds for data curation and sharing. Requests to access these datasets should be directed to hitten.zaveri@yale.edu.

## Supplementary material

SUPPLEMENTARY FIGURE S1 Estimates of magnitude squared coherence (MSC) evaluated in the gamma band for all possible pairs of electrode contacts within the hemisphere are shown as a function of inter-contact distance. Coherence estimates greater than 0.034 are significantly greater than 0 (p < 10^−5^)^28^. MSC estimates decrease considerably with distance between the contacts. We were interested in a measure of functional connectivity which was local to the electrode contact and chose to focus on a maximum intercontact distance of 5 cm to estimate band-related local functional connectivity (BRLFC).

## References

[B1] Andrzejak R. G. Zaveri H. P. Schulze-Bonhage A. Leguia M. G. Stacey W. C. Richardson M. P. (2023). Seizure forecasting: where do we stand? Epilepsia 64, S62–S71. 10.1111/epi.17546 36780237 PMC10423299

[B2] Bartolomei F. Lagarde S. Wendling F. McGonigal A. Jirsa V. Guye M. (2017). Defining epileptogenic networks: contribution of SEEG and signal analysis. Epilepsia 58 (7), 1131–1147. 10.1111/epi.13791 28543030

[B3] Bettus G. Wendling F. Guye M. Valton L. Régis J. Chauvel P. (2008). Enhanced EEG functional connectivity in mesial temporal lobe epilepsy. Epilepsy Res. 81 (1), 58–68. 10.1016/j.eplepsyres.2008.04.020 18547787

[B4] Bialonski S. Lehnertz K. (2013). Assortative mixing in functional brain networks during epileptic seizures. Chaos 23 (3), 033139. 10.1063/1.4821915 24089975

[B5] Blumenfeld H. Rivera M. Vasquez J. G. Shah A. Ismail D. Enev M. (2007). Neocortical and thalamic spread of amygdala kindled seizures. Epilepsia 48 (2), 254–262. 10.1111/j.1528-1167.2006.00934.x 17295618

[B6] Brazier M. A. (1972). Spread of seizure discharges in epilepsy: anatomical and electrophysiological considerations. Exp. Neurol. 36 (2), 263–272. 10.1016/0014-4886(72)90022-2 4559716

[B7] Brazier M. A. B. (1973). “Electrical seizure discharges within the human brain: the problem of spread,” in Epilepsy, its phenomenon in man. Editor Brazier M. A. B. (New York: Academic Press), 153–170.

[B8] Brazier M. A. B. (1972). “Interactions of deep structures during seizures in man,” in Mechanisms of synchronization in epileptic seizures. Editors Petsche H. Brazier M. A. B. (Vienna: Springer-Verlag), 409–424.

[B9] Burns S. P. Santaniello S. Yaffe R. B. Jouny C. C. Crone N. E. Bergey G. K. (2014). Network dynamics of the brain and influence of the epileptic seizure onset zone. Proc. Natl. Acad. Sci. U. S. A. 111 (49), E5321–E5330. 10.1073/pnas.1401752111 25404339 PMC4267355

[B10] Carter G. C. (1987). Coherence and time delay estimation. P Ieee 75 (2), 236–255. 10.1109/proc.1987.13723

[B11] Carter G. C. Knapp C. H. Nuttall A. H. (1973). Estimation of the magnitude-squared coherence *via* overlapped fast fourier transform processing. IEEE Trans. Acoust. Speech, Signal Process. 21 (4), 337–344. 10.1109/tau.1973.1162496

[B12] Chu C. J. Tanaka N. Diaz J. Edlow B. L. Wu O. Hämäläinen M. (2015). EEG functional connectivity is partially predicted by underlying white matter connectivity. Neuroimage 108, 23–33. 10.1016/j.neuroimage.2014.12.033 25534110 PMC4323839

[B13] Constable R. T. Scheinost D. Finn E. S. Shen X. Hampson M. Winstanley F. S. (2013). Potential use and challenges of functional connectivity mapping in intractable epilepsy. Front. Neurology 4, 39. 10.3389/fneur.2013.00039 23734143 PMC3660665

[B14] Dickten H. Porz S. Elger C. E. Lehnertz K. (2016). Weighted and directed interactions in evolving large-scale epileptic brain networks. Sci. Rep. 6, 34824. 10.1038/srep34824 27708381 PMC5052583

[B15] Eissa T. L. Schevon C. A. (2017). The role of computational modelling in seizure localization. Brain 140 (Pt 2), 254–256. 10.1093/brain/aww332 28137953 PMC6276902

[B16] Englot D. J. Hinkley L. B. Kort N. S. Imber B. S. Mizuiri D. Honma S. M. (2015). Global and regional functional connectivity maps of neural oscillations in focal epilepsy. Brain 138 (8), 2249–2262. 10.1093/brain/awv130 25981965 PMC4840946

[B17] Frei M. G. Zaveri H. P. Arthurs S. Bergey G. K. Jouny C. C. Lehnertz K. (2010). Controversies in epilepsy: debates held during the fourth International Workshop on Seizure Prediction. Epilepsy and Behavior E&B 19 (1), 4–16. 10.1016/j.yebeh.2010.06.009 20708976 PMC2943379

[B18] Geier C. Lehnertz K. (2017). Which brain regions are important for seizure dynamics in epileptic networks? Influence of link identification and EEG recording montage on Node centralities. Int. J. Neural Syst. 27 (1), 1650033. 10.1142/S0129065716500337 27377662

[B19] Goncharova I. I. Zaveri H. P. Duckrow R. B. Novotny E. J. Spencer S. S. (2009). Spatial distribution of intracranially recorded spikes in medial and lateral temporal epilepsies. Epilepsia 50 (12), 2575–2585. 10.1111/j.1528-1167.2009.02258.x 19674048

[B20] Goncharova I. I. Spencer S. S. Duckrow R. B. Hirsch L. J. Spencer D. D. Zaveri H. P. (2013). Intracranially recorded interictal spikes: relation to seizure onset area and effect of medication and time of day. Clin. Neurophysiol. 124 (11), 2119–2128. 10.1016/j.clinph.2013.05.027 23856192

[B21] Goncharova I. Alkawadri R. Gaspard N. Duckrow R. B. Spencer D. D. Hirsch L. J. (2016). The relationship between seizures, interictal spikes and antiepileptic drugs. Clin. Neurophysiol. 127, 3180–3186. 10.1016/j.clinph.2016.05.014 27292227

[B22] Gotman J. (1983). Measurement of small time differences between EEG channels: method and application to epileptic seizure propagation. Electroencephalogr. Clinical Neurophysiology 56, 501–514. 10.1016/0013-4694(83)90235-3 6194969

[B23] Gotman J. (1987). Interhemispheric interactions in seizures of focal onset: data from human intracranial recordings. Electroencephalogr. Clinical Neurophysiology 67 (2), 120–133. 10.1016/0013-4694(87)90034-4 2439288

[B24] Guye M. Regis J. Tamura M. Wendling F. McGonigal A. Chauvel P. (2006). The role of corticothalamic coupling in human temporal lobe epilepsy. Brain 129 (Pt 7), 1917–1928. 10.1093/brain/awl151 16760199

[B25] Holmes C. J. Hoge R. Collins L. Woods R. Toga A. W. Evans A. C. (1998). Enhancement of MR images using registration for signal averaging. J. Comput. Assisted Tomogr. 22 (2), 324–333. 10.1097/00004728-199803000-00032 9530404

[B26] Jiruska P. de Curtis M. Jefferys J. G. Schevon C. A. Schiff S. J. Schindler K. (2013). Synchronization and desynchronization in epilepsy: controversies and hypotheses. J. Physiology 591 (4), 787–797. 10.1113/jphysiol.2012.239590 23184516 PMC3591697

[B27] Khambhati A. N. Davis K. A. Lucas T. H. Litt B. Bassett D. S. (2016). Virtual cortical resection reveals push-pull network control preceding seizure evolution. Neuron 91 (5), 1170–1182. 10.1016/j.neuron.2016.07.039 27568515 PMC5017915

[B28] Kramer M. A. Kolaczyk E. D. Kirsch H. E. (2008). Emergent network topology at seizure onset in humans. Epilepsy Res. 79 (2-3), 17–86. 10.1016/j.eplepsyres.2008.02.002 18359200

[B29] Kramer M. A. Truccolo W. Eden U. T. Lepage K. Q. Hochberg L. R. Eskandar E. N. (2012). Human seizures self-terminate across spatial scales *via* a critical transition. Proc. Natl. Acad. Sci. U. S. A. 109 (51), 21116–21121. 10.1073/pnas.1210047110 23213262 PMC3529091

[B30] Kuhlmann L. Lehnertz K. Richardson M. P. Schelter B. Zaveri H. P. (2018). Seizure prediction - ready for a new era. Nat. Rev. Neurol. 14 (10), 618–630. 10.1038/s41582-018-0055-2 30131521

[B31] Lee H. W. Arora J. Papademetris X. Tokoglu F. Negishi M. Scheinost D. (2014). Altered functional connectivity in seizure onset zones revealed by fMRI intrinsic connectivity. Neurology 83 (24), 2269–2277. 10.1212/WNL.0000000000001068 25391304 PMC4277677

[B32] Lehnertz K. Brohl T. Wrede R. V. (2023). Epileptic-network-based prediction and control of seizures in humans. Neurobiol. Disease 181, 106098. 10.1016/j.nbd.2023.106098 36997129

[B33] Lieb J. P. Hoque K. Skomer C. E. Song X. W. (1987a). Inter-hemispheric propagation of human mesial temporal lobe seizures: a coherence/phase analysis. Electroencephalogr. Clinical Neurophysiology 67 (2), 101–119. 10.1016/0013-4694(87)90033-2 2439287

[B34] Lieb J. P. Babb T. L. J. Engel J. Darcey T. M. (1987b). “Propagation pathways of interhemispheric seizure discharges compared in human and animal hippocampal epilepsy,” in Fundamental mechanisms of human brain function: surgical treatment of epilepsy as an investigative resource. Editors Engel J J. Ojemann G. Lüders H. Williamson P. (New York: Raven Press), 165–170.

[B35] Mormann F. Lehnertz K. David P. Elger C. E. (2000). Mean phase coherence as a measure for phase synchronization and its application to the EEG of epilepsy patients. Phys. D. 144 (3-4), 358–369. 10.1016/s0167-2789(00)00087-7

[B36] Netoff T. I. Schiff S. J. (2002). Decreased neuronal synchronization during experimental seizures. J. Neurosci. 22 (16), 7297–7307. 12177225 10.1523/JNEUROSCI.22-16-07297.2002 PMC6757884

[B37] Nissen I. A. Stam C. J. Reijneveld J. C. van Straaten I. E. C. W. Hendriks E. J. Baayen J. C. (2017). Identifying the epileptogenic zone in interictal resting-state MEG source-space networks. Epilepsia 58 (1), 137–148. 10.1111/epi.13622 27888520

[B38] Palmigiano A. Pastor J. Garcia de Sola R. Ortega G. J. (2012). Stability of synchronization clusters and seizurability in temporal lobe epilepsy. PLoS One 7 (7), e41799. 10.1371/journal.pone.0041799 22844524 PMC3402406

[B39] Papademetris X. Jackowski A. P. Schultz R. T. Staib L. H. Duncan J. S. (2004). “Integrated intensity and point-feature non-rigid registration,” in Medical image computing and computer-assisted intervention Saint Malo. Editors Barillot C. Haynor D. Hellier P. (France: Springer), 763–770. 10.1901/jaba.2001.3216-763 PMC2869095 20473359

[B40] Park E. H. Madsen J. R. (2018). Granger causality analysis of interictal iEEG predicts seizure focus and ultimate resection. Neurosurgery 82 (1), 99–109. 10.1093/neuros/nyx195 28472428 PMC5808502

[B41] Rings T. Lehnertz K. (2016). Distinguishing between direct and indirect directional couplings in large oscillator networks: partial or non-partial phase analyses? Chaos 26 (9), 093106. 10.1063/1.4962295 27781446

[B42] Schevon C. A. Cappell J. Emerson R. Isler J. Grieve P. Goodman R. (2007). Cortical abnormalities in epilepsy revealed by local EEG synchrony. Neuroimage 35 (1), 140–148. 10.1016/j.neuroimage.2006.11.009 17224281 PMC1994936

[B43] Schindler K. A. Bialonski S. Horstmann M. T. Elger C. E. Lehnertz K. (2008). Evolving functional network properties and synchronizability during human epileptic seizures. Chaos 18 (3), 033119. 10.1063/1.2966112 19045457

[B44] Sinha N. Dauwels J. Kaiser M. Cash S. S. Brandon Westover M. Wang Y. (2017). Predicting neurosurgical outcomes in focal epilepsy patients using computational modelling. Brain 140 (2), 319–332. 10.1093/brain/aww299 28011454 PMC5278304

[B45] Sinha N. Joshi R. B. Sandhu M. R. S. Netoff T. I. Zaveri H. P. Lehnertz K. (2022). Perspectives on understanding aberrant brain networks in epilepsy. Front. Netw. Physiol. 2, 868092. 10.3389/fnetp.2022.868092 36926081 PMC10013006

[B46] Skrinjar O. (2002). Deformable models in image-guided neurosurgery. Yale University.

[B47] Smith E. H. Schevon C. A. (2016). Toward a mechanistic understanding of epileptic networks. Curr. Neurology Neuroscience Reports 16 (11), 97. 10.1007/s11910-016-0701-2 27662895

[B48] Smith E. H. Liou J. Y. Davis T. S. Merricks E. M. Kellis S. S. Weiss S. A. (2016). The ictal wavefront is the spatiotemporal source of discharges during spontaneous human seizures. Nat. Commun. 7, 11098. 10.1038/ncomms11098 27020798 PMC4820627

[B49] Spencer S. S. (2002). Neural networks in human epilepsy: evidence of and implications for treatment. Epilepsia 43 (3), 219–227. 10.1046/j.1528-1157.2002.26901.x 11906505

[B50] Spencer S. S. Goncharova I. I. Duckrow R. B. Novotny E. J. Zaveri H. P. (2008). Interictal spikes on intracranial recording: behavior, physiology, and implications. Epilepsia 49 (11), 1881–1892. 10.1111/j.1528-1167.2008.01641.x 18479398

[B51] Spencer D. D. Gerrard J. L. Zaveri H. P. (2018). The roles of surgery and technology in understanding focal epilepsy and its comorbidities. Lancet Neurology 17 (4), 373–382. 10.1016/S1474-4422(18)30031-0 29553383

[B52] Sritharan D. Sarma S. V. (2014). Fragility in dynamic networks: application to neural networks in the epileptic cortex. Neural Computation 26 (10), 2294–2327. 10.1162/NECO_a_00644 25058705

[B53] Staniek M. Lehnertz K. (2008). Symbolic transfer entropy. Phys. Rev. Lett. 100 (15), 158101. 10.1103/PhysRevLett.100.158101 18518155

[B54] Stone S. S. D. Park E. H. Bolton J. Harini C. Libenson M. H. Rotenberg A. (2022). Interictal connectivity revealed by granger analysis of stereoelectroencephalography: association with ictal onset Zone, resection, and outcome. Neurosurgery 91 (4), 583–589. 10.1227/neu.0000000000002079 36084171 PMC10553068

[B55] Terry J. R. Benjamin O. Richardson M. P. (2012). Seizure generation: the role of nodes and networks. Epilepsia 53 (9), e166–e169. 10.1111/j.1528-1167.2012.03560.x 22709380

[B56] Tomlinson S. B. Porter B. E. Marsh E. D. (2017). Interictal network synchrony and local heterogeneity predict epilepsy surgery outcome among pediatric patients. Epilepsia 58 (3), 402–411. 10.1111/epi.13657 28166392

[B57] Towle V. L. Syed I. Berger C. Grzesczcuk R. Milton J. Erickson R. K. (1998). Identification of the sensory/motor area and pathologic regions using ECoG coherence. Electroencephalogr. Clinical Neurophysiology 106, 30–39. 10.1016/s0013-4694(97)00082-5 9680162

[B58] Varotto G. Tassi L. Franceschetti S. Spreafico R. Panzica F. (2012). Epileptogenic networks of type II focal cortical dysplasia: a stereo-EEG study. Neuroimage 61 (3), 591–598. 10.1016/j.neuroimage.2012.03.090 22510255

[B59] Wang Y. Sinha N. Schroeder G. M. Ramaraju S. McEvoy A. W. Miserocchi A. (2020). Interictal intracranial electroencephalography for predicting surgical success: the importance of space and time. Epilepsia 61 (7), 1417–1426. 10.1111/epi.16580 32589284 PMC7611164

[B60] Warren C. P. Hu S. Stead M. Brinkmann B. H. Bower M. R. Worrell G. A. (2010). Synchrony in normal and focal epileptic brain: the seizure onset zone is functionally disconnected. J. Neurophysiol. 104 (6), 3530–3539. 10.1152/jn.00368.2010 20926610 PMC3007634

[B61] Wendling F. Chauvel P. Biraben A. Bartolomei F. (2010). From intracerebral EEG signals to brain connectivity: identification of epileptogenic networks in partial epilepsy. Front. Systems Neuroscience 4, 154. 10.3389/fnsys.2010.00154 21152345 PMC2998039

[B62] Wilke C. Worrell G. He B. (2011). Graph analysis of epileptogenic networks in human partial epilepsy. Epilepsia 52 (1), 84–93. 10.1111/j.1528-1167.2010.02785.x 21126244 PMC3200119

[B63] Yaffe R. B. Borger P. Megevand P. Groppe D. M. Kramer M. A. Chu C. J. (2015). Physiology of functional and effective networks in epilepsy. Clin. Neurophysiol. 126 (2), 227–236. 10.1016/j.clinph.2014.09.009 25283711

[B65] Zaveri H. P. Williams W. J. Sackellares J. C. Beydoun A. Duckrow R. B. Spencer S. S. (1999). Measuring the coherence of intracranial electroencephalograms. Clin. Neurophysiol. 110 (10), 1717–1725. 10.1016/s1388-2457(99)00136-4 10574287

[B66] Zaveri H. P. Duckrow R. B. Spencer S. S. (2000). The effect of a scalp reference signal on coherence measurements of intracranial electroencephalograms. Clin. Neurophysiol. 111 (7), 1293–1299. 10.1016/s1388-2457(00)00321-7 10880805

[B67] Zaveri H. P. Pincus S. M. Goncharova I. I. Duckrow R. B. Spencer S. S. (2008). Large scale brain networks in epilepsy. P Soc. Photo-Opt Ins. 7074, 70740T-T–10. 10.1117/12.801365

[B68] Zaveri H. P. Pincus S. M. Goncharova I. I. Duckrow R. B. Spencer D. D. Spencer S. S. (2009a). Localization-related epilepsy exhibits significant connectivity away from the seizure-onset area. Neuroreport 20 (9), 891–895. 10.1097/WNR.0b013e32832c78e0 19424095

[B69] Zaveri H. P. Pincus S. M. Goncharova I. I. Novotny E. J. Duckrow R. B. Spencer D. D. (2009b). A decrease in EEG energy accompanies anti-epileptic drug taper during intracranial monitoring. Epilepsy Res. 86 (2-3), 153–162. 10.1016/j.eplepsyres.2009.06.002 19632096

[B70] Zaveri H. P. Pincus S. M. Goncharova I. I. Novotny E. J. Duckrow R. B. Spencer D. D. (2010). Background intracranial EEG spectral changes with anti-epileptic drug taper. Clin. Neurophysiol. 121 (3), 311–317. 10.1016/j.clinph.2009.11.081 20075002

[B71] Zaveri H. P. Schelter B. Schevon C. A. Jiruska P. Jefferys J. G. R. Worrell G. (2020). Controversies on the network theory of epilepsy: debates held during the ICTALS 2019 conference. Seizure 78, 78–85. 10.1016/j.seizure.2020.03.010 32272333 PMC7952007

