# Supplementary figures and images for: Spatial and spectral structure of local functional connectivity of the background intracranial EEG in patients with focal epilepsy
Source: Front Netw Physiol. 2026 Jan 9;5:1441949. doi: 10.3389/fnetp.2025.1441949 (PMC12827612; doi:10.3389/fnetp.2025.1441949)

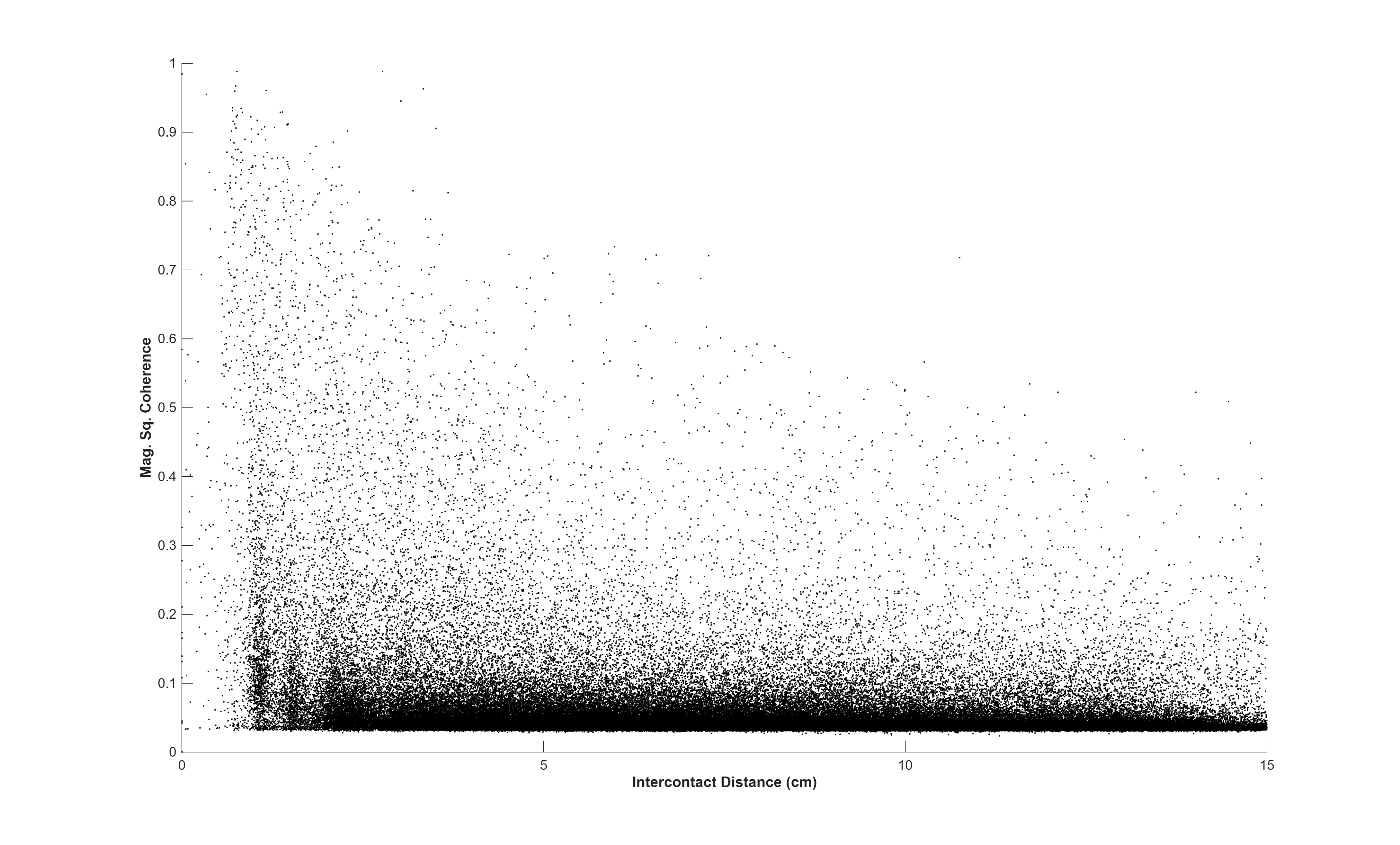

Supplement: Supplementary file 1 [file Image1.tif]
